# Quinoa in Ecuador: Recent Advances under Global Expansion

**DOI:** 10.3390/plants10020298

**Published:** 2021-02-04

**Authors:** Leonardo Hinojosa, Alex Leguizamo, Carlos Carpio, Diego Muñoz, Camilo Mestanza, José Ochoa, Carmen Castillo, Angel Murillo, Elena Villacréz, Carlos Monar, Nicolas Pichazaca, Kevin Murphy

**Affiliations:** 1 Institute for Biodiversity and Ecosystem Dynamics (IBED), University of Amsterdam, 1098XH Amsterdam, The Netherlands; 2 Comité Europeo Para la Formación y la Agricultura (CEFA), Guayas 22-46 y Venezuela, Lago Agrio EC210105, Ecuador; a.leguizamo@cefaonlus.it; 3 Facultad de Recursos Naturales, Escuela Superior Politécnica de Chimborazo, Panamericana Sur km 1 1/2, Riobamba EC060155, Ecuador; ccarpio@espoch.edu.ec; 4 Grupo de Desarrollo de Tecnologías para la Reducción y Racionalización de Agroquímicos, Riobamba EC060155, Ecuador; diegom91_@hotmail.com; 5 Facultad de Ciencias Pecuarias, Carrera de Ingeniería Agropecuaria, Universidad Técnica Estatal de Quevedo-(UTEQ), km 7 ½ vía Quevedo–El Empalme, Mocache, Los Ríos EC120509, Ecuador; cmestanza@uteq.edu.ec; 6 Instituto Nacional de Investigaciones Agropecuarias Estación Experimental Santa Catalina, Panamericana Sur Km 1, Quito EC171107, Ecuador or jbochoa@uce.edu.ec (J.O.); carmen.castillo@iniap.gob.ec (C.C.); angel.murillo@iniap.gob.ec (A.M.); elena.villacres@iniap.gob.ec (E.V.); 7 Facultad de Ciencias Agrícolas, Universidad Central del Ecuador, Jerónimo Leiton s/n y Av. La Gasca, Quito EC170521, Ecuador; 8 Facultad de Ciencias Agropecuarias, Recursos Naturales y del Ambiente, Campus Laguacoto II, Universidad Estatal de Bolívar, Vía Guaranda a San Simón, Guaranda EC020150, Ecuador; cmonar@ueb.edu.ec; 9 Asociación de Productores de Semilla y Alimentos Nutricionales Andinos-Mushuk Yuyay (APROSANAMY), Cañar EC030304, Ecuador; maycela@hotmail.com; 10 Sustainable Seed Systems Lab, Department of Crop and Soil Sciences, College of Agricultural, Human, and Natural Resource Sciences, Washington State University, Pullman, WA 99164-6420, USA; kmuphy2@wsu.edu

**Keywords:** *Chenopodium quinoa*, quinoa production, participatory plant breeding, quinoa processing

## Abstract

Quinoa is a highly diverse crop domesticated in the Andean region of South America with broad adaptation to a wide range of marginal environments. Quinoa has garnered interest worldwide due to its nutritional and health benefits. Over the last decade, quinoa production has expanded outside of the Andean region, prompting multiple studies investigating the potential for quinoa cultivation in novel environments. Currently, quinoa is grown in countries spanning five continents, including North America, Europe, Asia, Africa, and Oceania. Here, we update the advances of quinoa research in Ecuador across different topics, including (a) current quinoa production situation with a focus on breeding progress, (b) traditional seed production, and (c) the impact of the work of the nongovernment organization “European Committee for Training and Agriculture” with quinoa farmers in Chimborazo province. Additionally, we discuss genetic diversity, primary pests and diseases, actions for adapting quinoa to tropical areas, and recent innovations in quinoa processing in Ecuador. Finally, we report a case study describing a participatory breeding project between Washington State University and the Association of Andean Seed and Nutritional Food Producers Mushuk Yuyay in the province of Cañar.

## 1. Introduction

Quinoa (*Chenopodium quinoa* Willd.) is an annual crop from South America. Originally thought to be domesticated in the highland region of the Andes Mountains, a recent report suggests that quinoa domestication from its wild relative *Chenopodium hircinum* Schard. occurred independently in the Andean highlands and on the Chilean coast [1]. Five ecotypes have been used to classify quinoas: (1) valley = grown at 2000 to 3500 m above sea level (m a.s.l.); (2) altiplano = grown at high altitudes of more than 3500 m a.s.l.; (3) salares = grown in the salt flats of Bolivia and Chile; (4) sea-level = grown at low altitude; (5) subtropical or yungas = grown in humid valleys of Bolivia [2]. 

Quinoa is adapted to a wide range of marginal environments [3]. It possesses high salinity tolerance and is considered as a facultative halophyte crop [4,5]. Quinoa can tolerate drought [6] and has been shown to grow well in controlled high temperature environments [7,8,9]; however, the combination of drought and heat can considerably reduce seed yield [9,10]. Quinoa seeds are highly nutritious, containing high contents of fiber, vitamins such as ascorbic acid, alpha-tocopherol, thiamin, and riboflavin, and minerals. Quinoa protein quality is high as it contains the nine essential amino acids in significant amounts, though not always in sufficient quantities to be a complete protein source to consumers across all age groups [11]. Moreover, quinoa possesses phytochemicals such as phenolics and bioactive peptides; these components have demonstrated beneficial effects on metabolic, cardiovascular, and gastrointestinal health [12,13,14]. 

Due to the characteristics described above, quinoa production has been expanding rapidly outside of the Andean region of South America over the last decade [15,16]. Peru and Bolivia are the major quinoa producers in the world, responsible for 86,011 and 70,763 tonnes (t) in 2018, respectively [17]. Ecuador was considered the third largest quinoa producer globally [17]; however, the extent of recent quinoa expansion worldwide is difficult to measure and it is a challenge to accurately rank Ecuador in quinoa production compared to other countries. Ecuador, one of the 17 most megadiverse countries in the world, is a small country in South America, divided into three continental regions: Amazon, mountains and coast, and one maritime region, the Galapagos Islands. [18,19]. 

The aim of this article is to summarize the advances in quinoa research in Ecuador. We discuss diverse aspects of the current quinoa production situation, and focus on breeding progress, traditional seed production in the province of Bolívar, and the impact of the quinoa work of the nongovernmental organization (NGO) “European Committee for Training and Agriculture” (CEFA) with farmers in the province of Chimborazo. Potential quinoa pests and diseases, genetic diversity, strategies for adapting quinoa to tropical areas, and innovations in quinoa processing are reported. Moreover, we include a case study highlighting a participatory breeding project between Washington State University (WSU) and the Association of Andean Seed and Nutritional Food Producers Mushuk Yuyay (Asociación de Productores de Semillas y Alimentos Nutricionales Andinos Mushuk Yuyay (APROSANAMY)) in the province of Cañar. 

## 2. Quinoa Production in Ecuador

Quinoa has been cultivated from southern Colombia to the coast of southcentral Chile, including parts of northwest Argentina and some subtropical lowlands in Bolivia [20]. It was one of the main crops and food sources in Incan society and later was partially displaced by cereals such as barley and wheat that were introduced during the Spanish conquest [2]. According to historians, Ecuadorian quinoa was cultivated before and after the Spanish conquest and has been one of the most important food sources in the indigenous communities in the Ecuadorian highlands. Traditionally, quinoa is cultivated in Ecuador between 2400 to 3400 m a.s.l. in different production systems [21].

**Figure 1:**
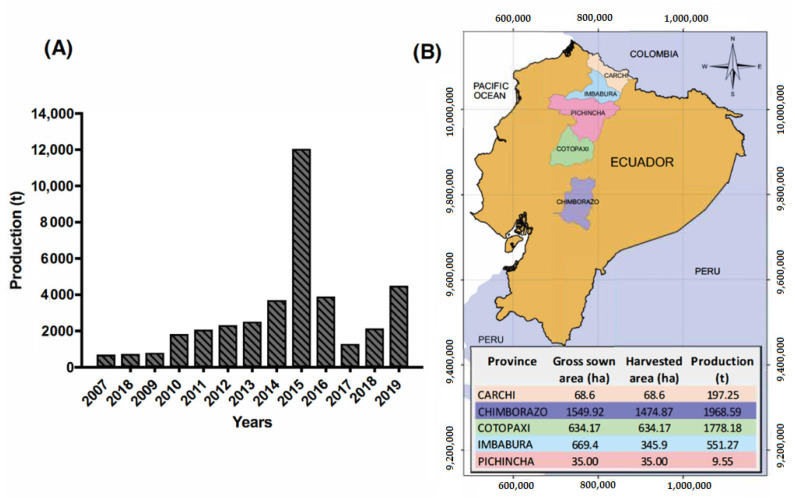
Quinoa production in Ecuador. (**A**) In the period from 2007 to 2019; (**B**) map including gross sown and harvested areas by province in 2019. Source: FAO and INEC-ESPAC [17,25].

Traditionally, Ecuadorian quinoa production was relatively constant and did not exceed 1000 t prior to 2010. In 2001, international partners started to work with Ecuadorian quinoa on different projects such as establishing producers associations and initiating participatory plant breeding projects [21,22,23]. These efforts resulted in slight production increases beginning in 2010. In 2015, quinoa production achieved a record high, reaching 12,000 t (Figure 1A). This increase was primarily due to government policies that encouraged quinoa production. The Ecuadorian Ministry of Agriculture created the project “Promotion of Quinoa Production in the Ecuadorian Sierra”, which delivered production kits that included seeds, fertilizers, and pesticides to hundreds of farmers. In 2015, the total production area reached 7800 hectares, partially fulfilling the goals of the project. However, the increased quinoa grain production did not have a readymade market; thus, a large amount of quinoa seed was stored for the following two years, and the fall in quinoa prices explains the drop in production in 2017 [16] (Figure 1A). International demand was high in 2015 and 2016, and quinoa production from Peru and Bolivia combined reached 180,000 t in 2015 [24].

In 2019, quinoa production increased again (4504 t) since many farmers’ associations, mainly in the provinces of Chimborazo and Cotopaxi, have stable international markets. NGOs such as Fundación Mujer y Familia Andina (Andean Woman and Family Foundation), European Committee for Training and Agriculture (CEFA), and the McKnight Foundation encouraged organic quinoa production in these two provinces [21,26]. The total area sown in 2019 was 2057 ha, led by Chimborazo province (1549 ha and 1968 t of production) and followed by Cotopaxi, Imbabura, Carchi, and Pichincha (Figure 1B). 

### 2.1. Quinoa Breeding in Ecuador 

In the 1980s, the national germplasm bank of quinoa was established at Ecuador’s National Institute of Agricultural Research (Instituto Nacional de Investigaciones Agropecuarias (INIAP)) with support from international cooperators and in consultation with Bolivian scientists. The first quinoa varieties, “INIAP Cochasqui” and “INIAP Imbaya”, were selected from the germplasm accessions, and in 1992 two low saponin content quinoa varieties, “INIAP Ingapirca” and “INIAP Tunkahuan”, were released [21]. Currently, “INIAP Tunkahuan” is the most widely planted variety among farmers, comprising 66% of total quinoa area with an average productivity of 1300 kg ha^−1^. In 2007, “INIAP Pata de Venado” was released due to its excellent adaptation to and production in high elevation environments (3000 to 3600 m a.s.l.) [21,24]. 

Quinoa hybridization started in Ecuador in 2008 at the INIAP Santa Catalina Experimental Station (Estación Experimental Santa Catalina (EESC)) at 3050 m a.s.l. using “INIAP Tunkahuan” and “INIAP Pata de Venado” in the crossing block. The primary breeding objective for this hybridization was to obtain early maturing lines, with large seed diameters and low saponin contents. In 2009, crosses were made using the Bolivian variety “Jacha” in order to obtain larger grain size (>2.2 mm). Evaluation of this population from the F_2_ to F_4_ was carried out at EESC using the pedigree method. Later, homogenous lines (F_5_–F_8_) were evaluated using participatory selection methodology in farmers’ fields in the provinces of Imbabura, Cotopaxi, Chimborazo, Cañar, and Bolívar. In 2019, the F_10_ lines LQEP4 and LQEP8, derived from the cross “INIAP Tunkahuan” × “INIAP Pata de Venado”, and lines EQ26 and EQ28, derived from the cross “INIAP Tunkahuan” × “Jacha” were selected. All four selected lines reached maturity in less than 170 days, had plant heights < 150 cm, were resistant to downy mildew, and yielded >1500 kg ha^−1^ (Table 1). Researchers at INIAP plan to release one or two new quinoa varieties from the four advanced lines in 2021 [27].

Additional hybridizations using accessions from the national germplasm bank of quinoa were made in 2016. The crosses ECU255 × EQ28 and ECU-248 × ECU-6205 produced F_5_ lines Q1 and Q2, characterized by high yield performances, early maturity, and short statured plants. In 2018, new crosses were made to obtain lines with low saponin contents, high yields, and different colors of seed such as black, red, and yellow. 

### 2.2. Participatory Research

Participatory research has been conducted in Ecuador for many years; for example, participatory quinoa breeding involved farmers from Cotopaxi, Chimborazo, and Cañar in order to select promising breeding lines and future varieties [22,23]. Participatory research has been carried out in the province of Bolívar. Here, we synthesize the main results from this participatory research. 

Bolívar province is located in the central region of Ecuador. It has a population of 200,000, of whom 60% live in rural areas, and agriculture is the main economic activity. The primary crops are corn (*Zea mays* L.), barley (*Hordeum vulgare* L.), bean (*Phaseolus vulgaris* L.), potato (*Solanum tuberosum* L.), faba bean (*Vicia faba* L.), Andean grains including Andean lupin “chocho” or “tarwi” (*Lupinus mutabilis* Sweet), and quinoa. 

Through a cooperative sustainability strategy with the Validation, Technology Transfer and Training Unit of Bolívar (Unidad de Validación, Transferencia de Tecnología y Capacitación-Bolívar (UVTT/C-B)) in Bolívar province, INIAP established local strategic alliances with the Ecuadorian Populorum Progressio Fund (Fondo Ecuatoriano Populorum Progressio (FEPP)), Ministry of Agriculture and Livestock (MAG), Instituto Técnico Superior Agropecuario “Tres de Marzo”, State University of Bolívar (Universidad Estatal de Bolívar (UEB)), and Alto Guanujo producers’ organizations. To initiate participatory research, the UVTT/C-B, selected two agronomic zones: (i) El Alto Guanujo (2900 to 3600 m a.s.l), its production system consisting of 98% potato and pasture, and (ii) Middle Zone (2500 to 2850 m a.s.l.), where the base crop is a monocropping of white soft corn associated with beans and to a lesser extent, wheat, barley, quinoa, and other legumes such as peas, lentil, and chocho.

Different people were involved in each zone because of diverse weather, social, culture and economic conditions. For instance, farmers from eight communities actively participated with researchers in a farmer field school in Alto Guanujo, the Comités de Investigación Agrícola Local (CIAL) “Progressio a la Vida”. In the Middle Zone, the main participants were students, professors, and researchers from UEB as well as INIAP technicians, and farmers. The result of this participatory research was the validation and technology transfer of new climate-resilient varieties of quinoa, potato, barley, faba bean, chocho, and pasture in the Alto Guanujo and quinoa, potato, bean, corn, wheat, barley, peas, lentils, and chocho in the Middle Zone. Moreover, it trained farmers to diversify their production systems by including these new varieties. Here, we summarize the main results for quinoa (Table 2).

### 2.3. Traditional Seed Production

When a variety has been generated, validated, and selected by the beneficiaries, it is essential for its dissemination to be designated “Quality Seed”, which must comply with specified physical, genetic, physiological, and health attributes [28]. Through a strategic alliance with MAG-Bolívar, UEB designed and implemented a small-scale seed research and production program to accelerate seed distribution to farmers. It was accredited as an official seed producer in November 2017 by the Ministry of Agriculture and Livestock under the rules and regulations of the Ecuadorian seed law. In collaboration with INIAP, MAG-Bolívar, and organizations of seed producers, UEB produces seed both in the conventional (“Certified” seed class) and artisanal models (Selected seed), whereas INIAP produces pure seed in “Basic” and “Registered” classes. 

To obtain high quality quinoa seeds, negative selection was applied in field seed production; thus, atypical plants, wild quinoas, and inferior plants were eliminated. In addition, UEB designed and implemented a participatory strategy to produce seed in the local area. Specifically, seeds were delivered to trained organizations of producers and agronomy students who planted the seeds and harvested the crop. After only one growing season, they returned to UEB twice the number of seed they originally received, thereby simultaneously increasing the volume of available seed and advancing its diffusion. The success of this seed program is linked to clearly determining quinoa seed variables of production, quality, quantity, continuity, and price.

### 2.4. The Nongovernmental Organization (NGO) “European Committee for Training and Agriculture” (CEFA) in Chimborazo

CEFA is a nonprofit, nonpolitical, and nondenominational NGO founded in Italy in 1972 and legally recognized in Ecuador through a technical cooperation framework agreement in 2009. In 2017, CEFA created the Inclusive and Sustainable Value Chains Program (Programa Cadenas de Valor Inclusivas y Sostenibles (PCV-IS)), funded by the European Union and the Italian Agency for Development Cooperation—AICS—whose objective is to create the quinoa chain in the province of Chimborazo, and within the framework of the MAG strategy, the popular and solidarity economy, and fair trade. The PCV-IS has three main components: (a) socio-organizational strengthening; (b) productive development; (c) associative marketing. In addition, implementing adaptation measures to climate change and including young people in the value chain are two transversa axes of the PCV-IS.

#### 2.4.1. Quinoa Chain in Chimborazo 

Chimborazo province is located in the central area of the Ecuadorian highlands (Figure 1B). It owes its name to the Chimborazo volcano that rises to 6268 m a.s.l. and is located 150 km south of the city of Quito in the Western Cordillera of the Andes. Chimborazo has a population of 524,004 inhabitants and is the ninth most populous province of Ecuador [29]. 

Cultivation of quinoa in the province of Chimborazo was promoted by the Fundación Escuelas Radiofónicas Popular del Ecuador (Popular Radio Schools of Ecuador Foundation (ERPE)) with the support of international cooperators. They led to the first export of quinoa with organic certification in 1998. Prior to 1998, quinoa was cultivated primarily for self-consumption. In 2003, the Corporación de Productores y Comercializadores Orgánicos Bio Taita Chimborazo (Bio Taita Chimborazo Organic Producers and Marketers Corporation (COPROBICH)) was formed, an organization that brought together producer families to comply with ERPE’s commercial agreements. Sumak Life Cía. Ltd. a joint venture between ERPE and COPROBICH, was formed in 2006. COPROBICH commercially dissociated itself from Sumak Life Cía. Ltd. in 2012 to enter the markets of France, Belgium, and Canada with certified organic quinoa and the Small Producers seal. In the same year, the Maquita Foundation began exporting certified organic quinoa with the fairtrade certification of the World Fair Trade Organization (WFTO).

Quinoa in Chimborazo is produced under the organic certification regulations of the EU and the USA. In economic terms, according to estimates by the PCV-IS based on information contained in the certification companies’ internal control systems, it represents a gross income of approximately USD 2,000,000 each year for 1500 producer families located in the cantons of Colta, Riobamba, Guamote, and Guano. According to the General Coordination of National Agricultural Information of the MAG, in 2019 the sale of quinoa to exporters was the main source of income for 60% of these families [30].

Sumak Life Cía. Ltd., COPROBICH, and Maquita Foundation together exported around seven hundred metric tons of quinoa grain every year, mainly to the European Union and North American markets, which corresponds to around 95% of the total production in Chimborazo province. The difference is divided between self-consumption and sale to local markets. Producers generally rotate quinoa with barley, wheat, faba bean, or a mix of vetches (*Vicia* spp.) and oats (*Avena sativa*) that are used as forage for their livestock.

#### 2.4.2. Main Actions of the PCV-IS in the Province of Chimborazo

##### Creation of a Technical Committee of Quinoa in Chimborazo

The objective of this technical committee is to analyze problems in production, transformation, commercialization, and consumption links of the quinoa chain in order to prioritize and articulate the actions of the direct and indirect actors. The direct actors in the quinoa chain are represented by Sumak Life Cía. Ltd., COPROBICH, Maquita Foundation, Sumak Tarpuy Corporation, and the Asociación de Producción y Comercialización de Productos Alimenticios Emprendedores Nutriandina (Association for the Production and Marketing of Food Products Entrepreneurs Nutriandina). Additionally, the indirect actors are represented by those who have support, regulation, investigation, or control functions in the chain, such as INIAP, MAG, Ecuadorian Agency for Quality Assurance in Agriculture (AGROCALIDAD), and the Polytechnic Higher School of Chimborazo (ESPOCH).

##### Promotion of Participatory Research and Links with the academy to Improve the Dynamics of Innovation in the Value Chain

Participatory research, led by INIAP, occurred in 18 field schools that were established in representative areas of the quinoa sector. Several studies were carried out on fertilization, intercropping systems, participatory selection of Chimborazo quinoa ecotypes, validation of the adaptation of quinoa advanced lines, and planting systems. In addition, ESPOCH implemented the project “*Diseño e Implementación del Proyecto de Producción, Transformación, Comercialización y Promoción de Consumo de la Quinua y sus Derivados”* (Design and Implementation of the Project for Production, Transformation, Commercialization and Promotion of Consumption of Quinoa and its Derivatives). This project involved seven departments and 60 researchers from different areas. Several undergraduate research and community linking projects were conducted as part of the project, engaging more than 260 students.

##### Identification and Validation of Three Species for Intercropping with Quinoa

**Figure 2:**
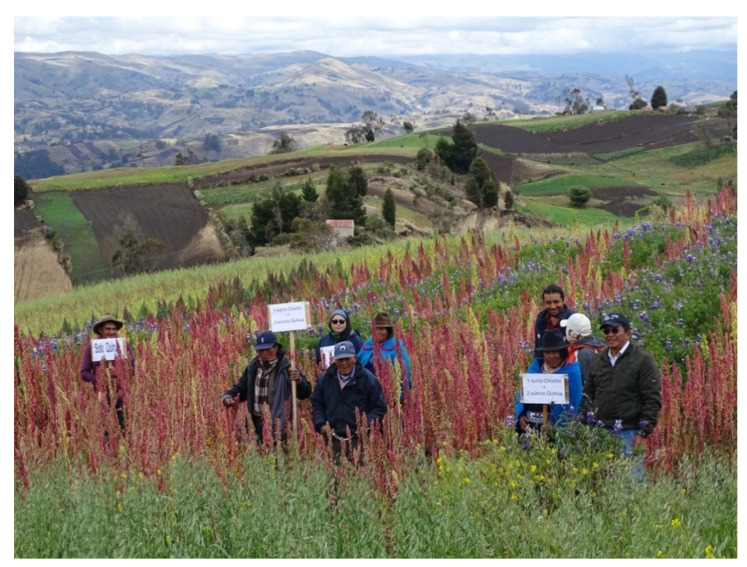
Intercrop system of chocho (*Lupinus mutabilis*) with quinoa. Comunidad Achullay, Guamote. Juan Yuquilema (farmer), Fausto Yumisaca (INIAP Chimborazo), Rodrigo Aucancela, and Galo Morocho (CEFA). Photo: A. Leguizamo.

Common vetch (*Vicia sativa*) was sown in an intercropped system at the time of quinoa hilling. After the quinoa was harvested, the vetch remained a remnant for the grazing of breeding animals. A faba bean variety, “INIAP-441 Serrana”, was sown in the system comprised of one row of faba bean x three rows of quinoa, for areas located above 3200 m a.s.l. in loamy to clay loam soils. Faba bean contributes nitrogen to the quinoa crop and is used for family consumption and occasionally for the marketing of surpluses. Finally, a chocho variety, “INIAP-450 Andino”, was planted in a system comprised of one row of chocho × three rows of quinoa for farms below 3200 m a.s.l. in sandy and sandy loam soils (Figure 2).

##### Use of Local Organic Matter Source for Soil Improvement

ESPOCH, through the Faculty of Sciences and the local government of Riobamba, will begin to recover the vegetable residues from local markets, compost them, and then supply the compost to quinoa farmers. Approximately 1000 t of compost per year are expected to be recovered from this initiative.

##### Development and Implementation of Adaptation Measures to Climate Change

Quinoa soils are vulnerable to water erosion during intense rains, especially fields with newly planted crops. Therefore, a transition from the traditional tillage system to one of minimum tillage, including cover crops such as vetch and direct seeding of quinoa, is required.

## 3. Pests and Diseases

### 3.1. Quinoa Pests in Ecuador

A formal study of insect pests affecting quinoa in Ecuador was conducted 30 years ago [31]. Species identification was performed by international experts at that time. Seven lepidopterans from the Noctuidae family were identified affecting quinoa. Cutworms damaging plantlets in the field such as *Agrotis deprivata* Walker and *A. ipsilon* (Hufnagel) were reported. The following leaf or grain chewers were also reported: *Peridroma saucia* (Hübner), *Dargida grammivora* Walker, *Spodoptera* sp., and two nonidentified species of *Copitarsia*. Two other lepidopterans were identified—*Scrobipalpula* sp. in the Gelechiidae family affected the leaves and an *Ephestia* sp. of the Pyralidae family damaged the stored quinoa seed. Two tachinid flies were found: *Incamyia* sp. was identified parasitizing both *Agrotis* species and *Elfia* sp. was a parasitoid of *Scrobipalpula* sp. Two species within the Coleoptera order were identified: the curculionid *Naupactus* sp., which eats quinoa leaves and grains, and *Oryzaephilus surinamensis* (L.) (Cucujoidea), which affects stored quinoa flour. *Liriomyza* sp. (Diptera: Agromyzidae) is a leaf miner reported in quinoa. The sap feeders *Paratanus yust* Young (Hemiptera: Cicadellidae) and *Proba sallei* (Stal) (Hemiptera: Miridae) were identified as affecting quinoa plants in the field. The same study reported the presence of a few natural enemies of quinoa pests—two tachinid flies were found: *Incamyia* sp. was identified as parasitizing both *Agrotis* species and *Elfia* sp. as a parasitoid of *Scrobipalpula* sp. [31].

Primary pests of quinoa are only briefly mentioned in crop management documents. One publication reports cutworms (*Agrotis* sp.) in young plants and birds are the most common pests [32]. Recently, between June and August 2020, an insect inventory was carried out to survey entomofauna associated with organic quinoa fields at six locations in the province of Chimborazo. The most abundant insects collected in this inventory were from the following orders: Hemiptera (one family), Coleoptera (three families), Lepidoptera (two families); parasitoid insects from the order Hymenoptera (one family), and predatory insects from the Neuroptera (one family) (Table 3). The main phytophagous insects observed on the plants belonged to the family Aphididae (Hemiptera), which attack the leaves, and Gelechiidae (Lepidoptera), which feeds on the inflorescence (Table 3). It is also important to point out the presence of parasitoids the of Braconidae family (Hymenoptera).

Insects of the family Cucurlionidae were the most abundant phytophagous insects found in the soil around the roots (Table 4). Cucurlionidae fed on the external parts of roots and did not bore into the roots. Additionally, a greater quantity of Annelidae (earth worms) was found in the plots from the lower elevation locations.

Insects from the following orders were collected at the lower elevation locations using yellow bowl pan traps placed 40 cm above the soil: Coleoptera (two families), Diptera (16 families), Hemiptera (four families), Hymenoptera (15 families), Lepidoptera (one family), and Thysanoptera (one family). In the traps of the plots in the location at the highest altitude, insects from the following orders were collected: Coleoptera (one family), Diptera (21 families), Hemiptera (three families), Hymenoptera with 10 families (Table 5), Neuroptera (one family), and Thysanoptera (one family).

The richness of individuals collected of Hymenoptera may indicate the good health of the plots sampled, since we know that individuals of this order are sensitive to the application of insecticides. In the plots from the lower altitude locations, most of the Hymenoptera belonged to Halictidae, Braconidae, Chalcididae, and Ichneuminidae, whereas most of the Hymenoptera in the plots from the highest altitude locations belonged to Crabronidae, Pteromalidae, Bethylidae, and Megaspilidae (Table 5).

### 3.2. Quinoa Diseases in Ecuador 

In most cultivated plants, diseases are major biotic constraints usually involving a wide range of pathogens. Unlike most crops, quinoa is in general a marginal host for plant pathogens. In Ecuador, neither viruses nor bacteria have been reported to infect quinoa [33], though this could be due to a lack of pathology research. *Cercospora* sp. and *Phoma* sp., the most common foliar fungi [34], are low damaging pathogens. In Ecuador, the most economically important quinoa disease is downy mildew (DM), caused by the oomycete *Peronospora variabilis*.

This low range of pathogens infecting quinoa in Ecuador agrees to some extent with the worldwide quinoa disease review reported by Danielsen et al. [35]. However, *Sclerotium rolfsii* and *Pythium zingiberum,* causing seedling diseases, and *Ascochyta hyalospora* and *Phoma exigua* var. *foveata*, causing ascochyta leaf spot and brown stalk rot, respectively, have not been reported in Ecuador. *Rhizoctonia solani* and *Fusarium* sp. causing damping off have been observed sporadically but are not considered threats for quinoa in Ecuador. 

#### 3.2.1. Downy Mildew

Downy mildew (DM) is the most damaging disease that threatens quinoa production worldwide [35]. In Ecuador, this disease is especially important in commercial monoculture of uniform varieties. The Department of Plant Pathology and the Quinoa Breeding Program of INIAP have been conducting research to identify and introgress durable resistance (DR) into quinoa DM, which is highlighted in this report.

*Peronospora variabilis*, previously classified as *P*. *farinosa* f. sp. *chenopodii*, was considered specific to quinoa; however, the pathogen has also been found infecting *Chenopodium album* and *C. murale* [36,37]. In Ecuador, isolates infecting *C. album* belong to the same population that infects quinoa and since *C. album* is a common weed of quinoa, the secondary host status of *C. album* is an important aspect to consider for DM management as well as in breeding for DR.

*P. variabilis* reproduces by heterothallic sexual reproduction [38], and two mating types appear to occur frequently, since oospores are massively produced on the leaves and carried by the seed [39,40]. In addition, oospores transmitted by seed are the primary pathogen inocula, and seed transmission is the main dispersion strategy for the pathogen to reach regions outside the Andes.

Studies of pathogenic diversity have shown that *P. varibilis* is a variable and adaptable pathogen [41]. Four virulence groups (Vs) of *P. varibilis* have been identified with respect to R-3 factors (R). V1 infects only susceptible lines, V2 infects susceptible lines and lines carrying R1, V3 infects susceptible lines and lines carrying R1 and R2, and V4 infects susceptible lines and lines carrying R1, R2, and R3. Pathogen evolution that has taken place in Ecuador is associated with single successive step mutations, which is in turn associated with the breeding initiatives carried out during the 1980s and early 1990s, which exploited the three R-factors, and R2 and R3 were released in varieties INIAP Ingapirca and INIAP Imbaya, respectively. Levels of resistance to DM at time of variety release were lower than 4 on a 0–9 scale, values located in the resistance fraction of the scale [21].

*P. varibilis* evolution is associated with mutation events. Although genetic recombination has not yet been evident, heterothallism is a potential mechanism by which the pathogen evolves beyond R-factor adaptation to include improved adaptation to environmental conditions and increased aggressiveness. Efficient seed transmission of the pathogen facilitates this potential mode of pathogen evolution. These epidemiological aspects are primarily associated with modern quinoa cultivation, in which cultivating genetically uniform varieties drives higher selection pressures on the pathogen. 

Development of strategies to search for DR to DM quinoa should be an important objective of modern breeding programs. Since R-factors are often ephemeral, screening for new sources of R-factors is not advisable. Therefore, non-race-specific resistance appears to be the most effective strategy to attain DR in the quinoa/downy mildew pathosystem. In Ecuador, “INIAP Tunkahuan” carries this type of resistance and has remained effective since variety release in 1992. Resistance of “INIAP Tunkahuan” can be considered durable since it aligns with the requirements for durable resistance as proposed by Johnson [42]. Thus, “INIAP Tunkahuan” has been hegemonically cultivated in commercial monoculture in a relatively large area, in conditions conducive to the disease, and for a long period of time (more than 25 years).

Levels of non-race-specific resistance were studied in four locations in Ecuador conducive to DM [22]. Resistance varied from low to high in lines without R-factors and in lines with defeated R-factors (residual resistance). Furthermore, lines carrying non-race specific resistance scored area under disease progress curve (AUDPC) values as low as lines carrying efficient R-factors, indicating that non-race-specific resistance can provide the same high level of protection as provided by R-factors. This resistance of non-race-specific type is quantitative in nature, and in other downy mildews, as in lettuce, this resistance is polygenic, with major and minor effects [43,44]. The high variation of AUDPC in lines with non-race-specific resistance suggests that this type of resistance is polygenic. In this study, lines were found with similar or higher levels of resistance than “INIAP Tunkahuan”, suggesting that sources of non-race-specific resistance are available in quinoa germplasm of the Quinoa Breeding Program at INIAP.

The early Ecuadorian experience in selection for DM resistance showed that breeders are interested in high levels of resistance, and by selecting lines with low disease severities, they are unconsciously favoring R-factors. However, they eventually also select high levels of non-race-specific resistance when lines carry similar disease severities to R-factors, which is the case of “INIAP-Tunkahuan”. Since R-factors resistance appears ephemeral, non-race-specific resistance is a more durable resistance strategy. Discarding the most susceptible and most resistant lines, as proposed by Parlevliet (1993) appears a practical strategy to favor selection of non-specific resistance and to select for DR to DM [45]. However, by discarding resistant lines, other promising traits and residual resistance can be inadvertently discarded. Therefore, to select non-race-specific resistance more efficiently, discard the most susceptible lines with the assistance of R-factors postulation to assess residual resistance in the field. This strategy is presently being implemented by the Quinoa Breeding Program of INIAP.

#### 3.2.2. Minor Diseases

Quinoa leaf spot caused by *Cercospora* sp is considered a minor disease in Ecuador [34]. Symptoms regularly develop late in the season on leaves of the upper part of the plant. Symptoms are characterized by round to oval and brown to grey lesions with diameters of less than 1 cm with darker brown reddish margins. Similar symptoms are also found in Ecuador on *Chenopodium album* [34]. This pathogen was also detected in the USA on quinoa, and the fungus was phylogenetically similar to *Passalora dubia*, which infects *C. album*; therefore, *P. dubia* is presently the taxonomical status of the causal agent of the quinoa leaf spot, known in the USA as *Passalora* leaf spot [46]. The incubation period of *P. dubia* of around 30 days [46] is significantly longer than that of *Cercospora* sp., which might explain its limited pathogenicity and why epidemics appear late in the quinoa growing season. 

Eye-like stalk spot caused by *Phoma* sp. is also a disease that affect quinoa late in the cropping season. Symptoms are gray spots with diameters of 1–3 cm surrounded by brown to dark margins. Dark pycnidia of the pathogen become regularly visible in the gray background of the lesion [34,35]. In conducive conditions, lesions can join and cover a considerable area of stems. Lesions can also be produced by mechanical injuries such as those caused by hailstorms.

## 4. Genetic Diversity 

Ecuadorian quinoa belongs to the valley ecotype. This quinoa ecotype is characterized by tall, late-maturing plants with high seed yield potentials and biomass accumulation [21]. Seed yield is negatively affected when the photoperiods exceeds 14 h in high temperature environment [2,47]. Currently, the quinoa germplasm at INIAP contains 608 accessions—283 from Ecuador (46%) and the rest from other countries, primarily Peru and Bolivia [48]. 

In recent decades, significant genetic erosion of quinoa landraces has been observed in the field. For instance, 30 years ago, variation in quinoa grain color was easy to find, including colors such as white, cream, brown, yellow, red, pink, and black. Currently, just white- or cream-colored quinoa grains are grown by most farmers, due to market demand and eating patterns in production areas [49]. A core quinoa collection using 469 quinoa accessions from the Ecuadorian quinoa germplasm bank was evaluated, and discriminating traits such as downy mildew reaction, seed color, days to flowering, and seed yield differentiated four genetic groups [50].

Traditionally, Ecuadorian quinoa is positioned to have limited genetic diversity, with Altiplano (Peru–Bolivia) quinoa indicated as the most probable point of introduction for Ecuadorian accessions [20]. This evidence came from the molecular and genetic diversity studies where just two Ecuadorian quinoa accessions were included [51,52]. Recently, a molecular characterization of diversity of Ecuadorian quinoa was conducted using 15 species-specific SSR markers on 84 quinoa accessions [53]. The results showed that Ecuadorian quinoa was highly diverse—196 alleles were detected, and the genetic heterozygosity (*H_E_*) was 0.71. Three subgroups were determined by a phenetic analysis—one group corresponded to the “INIAP Tunkahuan” variety that has been exposed to intense inbreeding and selection. The three groups included quinoa from the seven provinces where seed was collected, and indicated that ancestral landrace populations had been disseminated throughout Ecuador mainly due to farmers’ seed interchange.

### Wild Quinoa Relatives 

Wild quinoa is known as “ashpa quinoa” in the Quichua language or “ajara” in the Aymara language. The wild quinoa in Ecuador has been poorly studied; however, there is a report that a free-living (wild, weedy, spontaneous) quinoa population named as *C. quinoa* ssp. *milleanum* Aellen or var. *melanospermum* is distributed mainly in the mountains of Ecuador [54]. There is a hypothesis that *C. quinoa* ssp. *milleanum* could be closely related to the North American tetraploid species *C. berlandieri* subs. *zschachkei* and the South American tetraploid species *C. hircinum* ssp. *catamarcense* [54,55]. 

A phylogenetic study focused on the amplification and sequences of gene rpoB in 32 wild quinoa accessions collected from four provinces including Carchi, Imbabura, Cotopaxi, and Chimborazo showed high inter- and intrapopulation genetic diversity across the accessions [56]. Accessions from Carchi and Imbabura had 77.4% of the variability and genotypes from Cotopaxi and Chimborazo had 70.2%. In this study, all the wild quinoas were designated as *C. quinoa*; this description resulted in a misunderstanding between the cultivated quinoa and the wild quinoa in Ecuador. Thus, it is necessary to conduct more molecular studies including *C. berlandieri*, *C. hircinum,* and quinoa varieties as controls to understand the evolutionary origin of Ecuadorian quinoa. 

**Figure 3:**
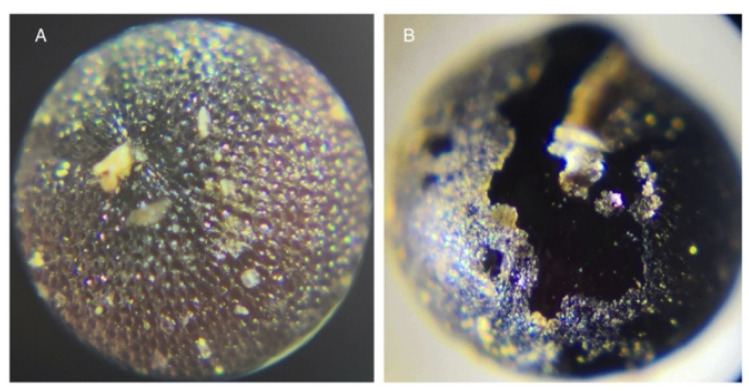
Wild quinoa relatives’ seed surfaces. (**A**) Probably *C. quinoa* ssp. *milleanum,* or *C. berlandieri*, or *C. hircinium*, with a pitted surface. (**B**) Probably *C. album*, with a smooth surface. Magnitude: 60×. Photos: L. Hinojosa.

A collection of wild quinoas in Carchi and Chimborazo showed a significant difference in seed morphology (unpublished data). The seed gathered from farmers’ fields in Carchi showed a pitted surface (Figure 3A). It is likely that these plants are *C. berlandieri*, *C. hircinum*, or the *C. quinoa* ssp. *milleanum* [57]. On the other hand, the seed collected in Chimborazo had a smooth surface (Figure 3B). It is possible that this species belongs to the *C. album* complex, which has a cosmopolitan distribution around the world [58]. Molecular studies are necessary to confirm this hypothesis.

Interspecific hybridizations in nature between *C. quinoa* and *C. berlandierie, C. hircinum,* and *C quinoa* ssp. *milleanum* have been reported [54,55,59]. Plant breeders have intentionally created interspecific crosses (simple and reciprocal) in order to recombine favorable traits (e.g., heat tolerance) present in different species and to concentrate them in the selected offspring [60]. Studies are required to identify if wild quinoas in Ecuador can cross with cultivated quinoas; this feature could be beneficial to breeding programs. 

## 5. Quinoa Adaptation to Tropical Areas 

**Figure 4:**
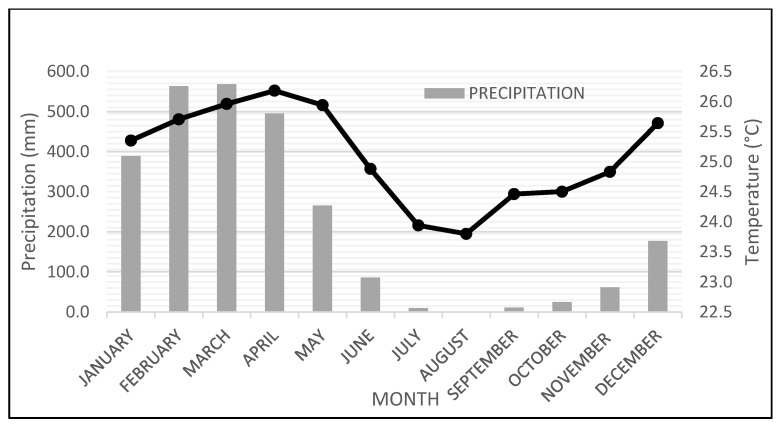
Average monthly precipitation and temperature for the last ten years (2010–2019) in the Quevedo area.

Of the five quinoa ecotypes described above, the valley ecotype is predominantly produced and consumed in Ecuador [61]. Little information exists on the adaptation of quinoa to coastal or tropical areas in Ecuador. In 2014, Peralta et al. evaluated the adaptability of 269 quinoa accessions from the quinoa germplasm existing at INIAP, under the agro-ecological conditions of the Santa Elena Peninsula; however, all accessions evaluated proved to be poorly adapted to the area mainly due to high temperature [62]. The interest in producing quinoa on the coast of Ecuador arises from the increase in consumption worldwide, high international prices [63], and the experiences of coastal production in Chile [64] and Peru [65]. In addition, knowing quinoa’s great capacity for adaptation [3] makes the northern area of the province of Los Rios (Quevedo) a suitable niche for the production of rainfed quinoa (Figure 4). 

In collaboration with other researchers, multiple trials were established in 2017 to evaluate quinoa genotypes from the coast and south of Chile, Argentina, and Ecuador. In the first trial, 21 accessions were sourced from Chile and Argentina, and grown from August to December 2017 at the research station “La Maria” of the State Technical University of Quevedo located in Mocache, Quevedo [66]. The results demonstrated the feasibility of quinoa production in this area. Days to maturity ranged from 90 to 143 days after sowing and yield ranged from 346 to 470 g m^−2^. Several colors in the grain such as yellow, white, red, and black were described. 

In the second trial at Mocache, Quevedo, genotypes from Ecuador, Chile and Argentina were surface-seeded and grown from June to September 2019 [67]. Although this trial was affected by poor germination, the genotype O-10 (red leaf color) performed well, with a crop cycle of 110 days, a grain yield of 3723 kg ha^−1^, a harvest index of 0.41, and plant height of 90 cm.

Follow-up investigations were carried out (data not yet published), which demonstrate that the best genotype (O-10) under these conditions, needs 1300 accumulated degree days to finish its cultivation cycle in 110 days. In addition, based on the efficient use of nitrogen in rainfed conditions, it is recommended to apply 62 kg N ha^−1^ if the objective is to achieve maximum N use efficiency. However, if the objective is to achieve maximum grain performance, it is recommended to apply 110 kg N ha^−1^ to achieve a grain yield of 4700 kg ha^−1^. This research also showed that an increase in the nitrogen rate from 0 to 200 kg ha^−1^ results in an increase in protein in the grain from 13% to 17%.

Currently, at the State Technical University of Quevedo (Universidad Técnica Estatal de Quevedo), research continues on adapting the red O-5 genotype and performing hybridizations with the cream-white O-3 genotype to obtain quinoa populations with adaptation criteria of the O-3, but with certain characteristics of O-5. Finally, it should be noted that our small collection of quinoas has shown the feasibility of being able to carry out two cultivation cycles a year, the first cycle from May to August and the second cycle from September to December. January to April is reserved for maize production because none of our quinoa genotypes will tolerate the excessive humidity that is generated during the first months of the year (Figure 4).

## 6. Quinoa Processing and Agroindustry 

Quinoa was widely used in South America during pre-Hispanic times; however, after the Spanish conquest, its cultivation and consumption were reduced to small areas scattered in mountainous areas of the Andes [32]. Since then, the grain was scarcely known or commercialized; however, the “re-discovery” of quinoa resulted in an explosion of its consumption, especially in Europe and the United States, in recent decades. This growth is largely a result of the high nutritional value of its gluten-free seeds and leaves, which have a moderate protein content (>15%), a good balance of all essential amino acids, dietary fiber, lipids, carbohydrates, vitamins, and minerals, and low glycemic index when consumed [13,68]. Functional compounds in quinoa such as polyphenols, phytosterols, and flavonoids have also been found to have important nutraceutical benefits [13,69]. 

### Quinoa Transformation

The Ecuadorian agroindustry processes innovative quinoa products for culinary, pharmaceutical, and cosmetic uses. Farmers and agribusiness enterprises that consider only grain production without the application of transformation technologies downgrade the commercial value of quinoa [21]. Several public and private institutions, universities, and research centers in Ecuador are currently developing improved varieties, including thoroughly characterizing the physico-chemical and nutritional traits, protein and amino acid contents, fatty acids, vitamins and minerals, phytohormones, antioxidants, phytosterols, and dietary fiber, as well as the identification and use of saponins [70,71,72,73]. Additionally, applications of thermal processes and bioprocesses have been studied in order to increase mineral bioavailability, the level of acceptability, and the nutraceutical value of quinoa grain [74].

The range of quinoa products that are produced and marketed in Ecuador include: whole grain, quinoa flour, quinoa flour mixtures with oats or amaranth, baby food porridge, granola, energy bars, soft drinks, quinoa expanded as breakfast cereal, biscuits, alfajores (a traditional dessert), and quinoa bread with substitution percentages reaching 30% [61,75]. On a smaller scale, and only in warehouses of products produced by farmers (fair trade), noodles are sold with some percentages of substitution. The Food World Programme (WFP), PANN-2000 (Programa de Alimentos), and “Aliméntate Ecuador” (“Feed Ecuador”) established as a mandate that both porridge and drinks for children must include quinoa as a raw material in their formulas [21]. Currently, solid fermentation processes are being tested to obtain vegetable meat and technologies are being optimized to produce milk- and yogurt-type drinks, products desired by children, adolescents, and adults, with national and international market potential among subjects with gluten allergy problems [75]. Additionally, precooked quinoa facilitates quinoa preparation and consumption in schools and assistance centers. The product can be seasoned with vegetables and can be introduced without much difficulty in various markets in Europe and the United States.

Quinoa leaves can be used as a nutritious vegetable, as they contain carotenoids, iron, and other micronutrients. The phytochemical profile shows that the leaves (60–80 days of cultivation) have low saponin contents. Their nutritional profile surpasses the grain, and they possess interesting potential in nutrition and food. Quinoa leaves can be used in the preparation of salads, soups, main dishes, and as raw material to enter the food industry, which wants to offer a continuous movement of new products in order to remain in the market, thereby contributing to the change of the production matrix [76,77]. 

It is worth highlighting the collaborations that private industry establishes with farmers capable of supplying large volumes of grain. Manufacturers in the food sector establish contracts with farmers. Contract farming is opening a very interesting way to consolidate the sustainability of the value chain [21]. Producers in the province of Chimborazo focus their attention on the production of organic fair-trade quinoa for traditional food applications in those areas less appropriate for intensification. However, Ecuadorian farmers face competition from other countries, as new varieties and models pave the way for large-scale production under different conditions.

## 7. Participatory Plant Breeding Project between WSU and APROSANAMY (Case Study) 

The Association of Producers of Seeds and Nutritious Andean Foods Mushuk Yuyay (APROSANAMY) was formed in 1994, with the main objective to focus on seed production of Andean crops such as barley, quinoa, faba bean, amaranth, and peas. In 2004, APROSANAMY started to add value to their production—for example, in the processing of barley and quinoa flour. In 2018, a new processing and storage facility was built to allow for the expansion of production. Currently, APROSANAMY has 69 producers from 18 communities from the cantons Cañar, Suscal, and El Tambo in Cañar province [78]. The province of Cañar, with a population of 281,396 inhabitants, is located between the provinces of Chimborazo and Azuay, in southcentral Ecuador [29].

Quinoa researchers at Washington State University (WSU) were first connected with APROSANAMY through Alan Adams, a former Peace Corps volunteer who worked in Quilloac, Cañar province, from 1967 to 1969. In May 2013, Alan received an email message from Nicolas Pichazaca requesting that he join in the work of APROSANAMY. Alan began researching, writing grants, and reaching out to potential collaborators. Based on a short article on quinoa in National Geographic that his wife Paulette read about WSU quinoa research, Alan contacted researcher Kevin Murphy and his Ecuadorian PhD student, Leonardo Hinojosa [79]. This began a formal arrangement between APROSANAMY and WSU to collaborate on quinoa and barley research. After initial discussions between Murphy and Hinojosa of WSU and Nicolas Pichazaca, Jose Luis Pichazaca, and Antonio Guaman of APROSANAMY regarding collaboration strategies with quinoa breeding, a two-pronged approach was implanted, including (a) evolutionary participatory breeding (EPB), and (b) evaluations of homogeneous cultivars and advanced breeding lines. 

The first strategy (EPB) included sharing a diverse breeding population to stimulate a participatory selection approach [80]. This approach involved selection from diverse bi-parental populations by APROSANAMY farmers and researchers, as well as natural selection in the different environments of Cañar, utilizing an evolutionary participatory breeding (EPB) approach [81,82]. EPB combines evolutionary breeding through natural selection with participatory selection by farmers and breeders to develop populations adapted to locally prevalent diseases and abiotic pressures [83,84]. These populations retain a significant amount of genetic variation, allowing the population to rapidly evolve in response to climate change and the resulting unpredictable fluctuations in environmental conditions [85,86]. Recent shifts in rainfall patterns and climate instability are key concerns voiced by Kichwa Kañari farmers.

Population development proceeded in two distinct phases. Phase I of the EPB approach focused on population development that incorporated traits of importance to Kichwa Kañari farmers. Based on multiple discussions between WSU and APROSANAMY, traits such as (a) early maturity to mitigate problems with preharvest sprouting due to increasingly unpredictable late season rains, (b) large seed size, (c) seed yield, and (d) red leaf color emerged as particularly important. Leonardo Hinojosa crossed cultivars such as “Titicaca” and “Puno”, and the red-leafed breeding line “3964” (developed collaboratively by researchers at Brigham Young University and WSU) with more regionally adapted varieties such as “Pasankalla”, a cultivar widely grown in Peru, and “CICA”, an Altiplano cultivar from Peru that was shared kindly by Juan Antonio Gonzales from Argentina. Additionally, the “Titicaca”, “Puno”, “3964”, “QQ74”, “Colorado 407D”, “Cahuil”, and “Kaslaea” varieties were grown as control cultivars next to the populations (Table 6). 

**Figure 5:**
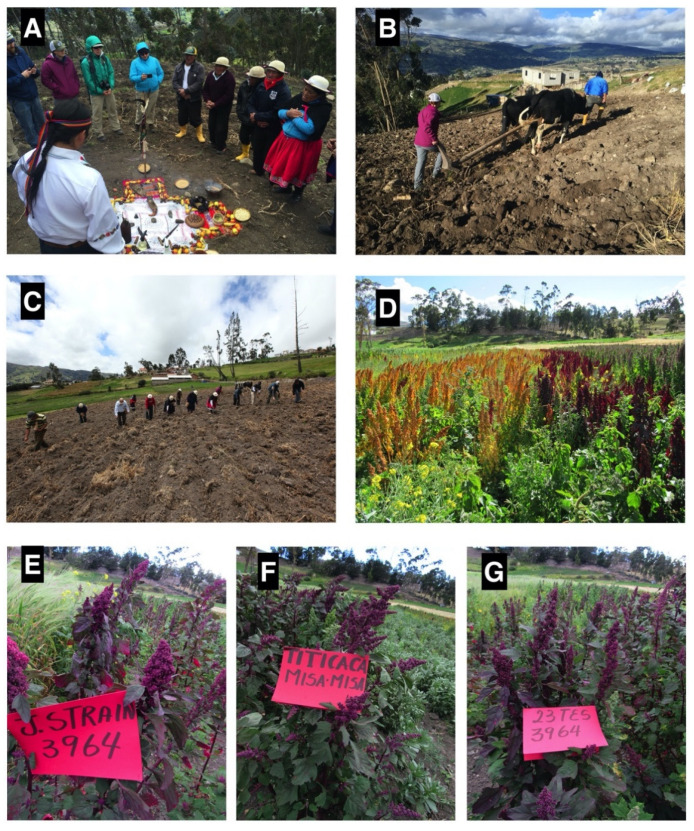
Asociación de Productores de Semillas y Alimentos Nutricionales Andinos Mushuk Yuyay (APROSANAMY) and Washington State University (WSU) activities. (**A**) Kañari planting ceremony; **(B)** field preparation, plowing (Macy Hagler, WSU student); (**C**) planting quinoa and barley with WSU students; (**D**) Line 3964 (red) and Colorado 407D (yellow); (**E**–**G**) introduction of new genetic variation (red color leaves). Photos by: L. Hinojosa, N. Pichazaca, and J. Kellogg.

These Phase I populations were planted by an APROSANAMY-WSU team in 2016 (Figure 5C) and have been replanted each year since then. By March 2018, Nicolas Pichazaca had selected the “Puno/Pasankalla” population and the “Kaslaea”, “QQ74”, “Colorado 407D”, and “3964” cultivars (Figure 5D), which were the most promising genotypes mainly due to early maturity, large seed size, and leaf color. These will continue to be grown in the future.

Phase II crosses continued to introduce new genetic variations designed to introgress traits such as heat tolerance (“Pison” and “QQ74”) [8], early maturity (Japanese Strain, “Titicaca”) red leaf color (“3964” and “MisaMisa”), and seed yield (“Titicaca”, “Kaslaea”) into adapted varieties such as “MisaMisa”, “Pasankalla”, and “INIAP Tunkahuan” (Figure 5E–G and Table 6).

The second strategy focused on testing pureline varieties and breeding lines. Fifty lines from the F_6_ population (“Cahuil”/”PI 510534”), created by former WSU graduate student Adam Peterson, were planted in Cañar in February 2019. The parents from this population include “Cahuil”, a variety that originated from Chile with good adaptation to Washington State, and “PI 510534”, an accession from National Plant Germplasm System of USDA that originated from the valley of Peru. Unfortunately, the seed germination from the lines was very low and drought and frost conditions caused the loss of the trial; however, new plantings are planned in the future with the same lines.

In October 2019, Kevin Murphy, Nicolas Pichazaca, Cristina Ocaña Gallegos (a new graduate student at WSU) planted 37 quinoa lines from the previous year’s selections. The trial was affected by high pressure of downy mildew and lines derived from “Puno”/”Pasankalla” showed high resistance to this pathogen.

In Table 7, we detail the timeline of the collaboration between APROSANAMY and WSU from 2015 to 2020. It is necessary to emphasize that in addition to the work on quinoa, WSU and APROSANAMY have been working in parallel with barley—including malting barley and hulless food barley. Currently, there are five promising lines under evaluation. Lines 13WA146-7, 13WA-146.3, and 13WA-146.1 were selected for their elongated grain shapes suitable for barley flour, “machika”, and pleasant smells during the manufacturing-grinding process; lines 13WA-146-10 and 13WA-126.6 were selected for “Arroz de Cebada” (“Barley Rice”), a traditional dish in the Ecuadorian highlands.

The next step in the quinoa EPB is to plant the selected quinoa lines at different locations and altitudes in the province of Cañar to evaluate yield performance and disease resistance. Moreover, APROSANAMY is interested in introducing the color panicle quinoa lines such as red, yellow, and orange close to archaeological sites where tourists are keen to see quinoa diversity.

## 8. Conclusions

Ecuador was considered the third largest quinoa producer globally, but with more recent expansion of this crop in other countries it is hard to assume that this statement is still true. The current quinoa production in Ecuador is around 4500 t per year and the Central provinces (Chimborazo and Cotopaxi) are the major producers. Participatory research has been carried out on quinoa for many years, mainly in the province of Bolívar. The National Agricultural Research Institute of Ecuador (INIAP) has released all the Ecuadorian quinoa varieties and new varieties are expected to be released soon from its hybridization program. The European Committee for Training and Agriculture (CEFA) has been working in the province of Chimborazo to support quinoa production; its main input was to develop a quinoa technical committee. On the other hand, new insects have been reported in quinoa fields, many of which are predators and parasitoids. Downy mildew (*Peronospora variabilis*) is the only damaging quinoa disease in Ecuador and durable resistance is the main objective of the quinoa breeding program at INIAP.

Ecuadorian quinoa is highly diverse contrary to previously thought. Wild quinoa has been poorly studied in Ecuador and there are no strong molecular studies showing its origin and identification; thus, new molecular works are necessary and must include wild quinoa and more landraces. On the other hand, new initiatives have begun to adapt quinoa to tropical coastal areas. Great progress is occurring in the province of Los Rios with the start of a new quinoa hybridization program at the State Technical University of Quevedo. In addition, Ecuador has developed different quinoa transformation strategies to link farmers and agribusiness enterprises, and Ecuadorian universities have conducted several research studies on nutrition and food sciences in the last five years.

A great effort has been made in the last five years to increase quinoa diversity in the Cañar province through an evolutionary participatory breeding project between Association of Producers of Seeds and Nutritious Andean Foods Mushuk Yuyay (APROSANAMY) and Washington State University (WSU). New quinoa colors, bigger seed size, and different panicle shapes have been introduced as well as early maturing quinoa. These new traits will help Cañar quinoa producers to achieve higher grain production and avoid late season rains and unpredictable droughts.

The current information presented in this article suggests that quinoa research and production in Ecuador is on the right track; however, it will be difficult to compete with the big technologies of countries such as China, Australia, and Spain where quinoa production has been recently rebounding. As a country, Ecuador needs to reinforce local consumption and internal demand for quinoa.

## Figures and Tables

**Table 1:** Average yield, plant height, days to harvest, and downy mildew severity of four promising quinoa lines evaluated during 2015–2019 in different locations in Ecuador.

Quinoa Line	Yield (kg ha^−1^)	Plant Height (cm)	Days to Harvest	Mildew Severity ^1^
LQEP4	1559	130	162	3
LQEP8	1708	125	160	3
EQ26	1522	121	161	5
EQ28	1508	120	160	4
INIAP Tunkahuan	1330	152	180	4

^1^ Severity scale of mildew caused by *Peronospora variabilis*: 1 to 3, first lower third of the plant (35%); 4 to 6, second middle third (35%); 7 to 9, last upper third of the plant (30%).

**Table 2:** Results of the participatory research on quinoa at two agronomic zones in the province of Bolivar, Ecuador.

Zone	Period	Variety	Selection Criteria	Comments
Alto Guanujo	2005–2016	“INIAP Pata de Venado”	Early, adapted to high altitude, resistant to freezing, wind, and mildew. Good flavor in soup. Used in rotation with potatoes.	Variety named “Pata de Venado” (Deer leg) (because deer are light, fast, and can resist cold, wind, and drought)
Middle Zone	2005–2019	“INIAP Tunkahuan Line ECU-6717”	Moderately early, tolerant to mildew, and resistant to lodging. The grain is white and sweet. Excellent for soups, flour, cookies.	Variety was grown in an intercrop system with corn “INIAP 111”, local corn varieties, chocho, and lentils

**Table 3:** Families of insects collected in destructive sampling of quinoa plants. The families are ordered in relation to abundance (from highest to lowest).

Order/Family	Functional Role
Hemiptera: Aphididae ^1^	Phytophagous
Coleoptera: Curculionidae	Phytophagous
Lepidoptera: Gelechiidae	Phytophagous
Coleoptera: Elateridae	Phytophagous
Coleoptera: Latriididae	Phytophagous
Lepidoptera: Arctiidae	Phytophagous
Hymenoptera: Braconidae	Parasitoid
Hemiptera: Aphididae ^1^	Phytophagous
Neuroptera: Chrysopidae	Predator
Lepidoptera: Noctuidae	Phytophagous

^1^ Different morphospecies.

**Table 4:** Insect families collected in soil blocks of quinoa plants removed from plots. The families are ordered in relation to abundance (from highest to lowest).

Order/Family	Functional Role
Coleoptera: Curculionidae ^1^	Phytophagous
Coleoptera: Sthaphylinidae	Predator
Coleoptera: Tenebrionidae	Detritivore
Lepidoptera: Noctuidae	Phytophagous
Himeptera: Aphidae	Phytophagous
Dermaptera: Anisolabididae	Detritivore, predator
Coleoptera: Curculionidae ^1^	Phytophagous

^1^ Different morphospecies.

**Table 5:** Families of Hymenoptera collected in yellow bowl pan traps in the province of Chimborazo. The families are ordered in relation to abundance (from highest to lowest).

Family	Functional Role
Halictidae	Pollinator
Braconidae	Parasitoid
Chalcididae	Parasitoid
Ichneumunidae	Parasitoid
Crabronidae	Predator
Pteromalidae	Parasitoid
Bethylidae	Parasitoid, predator
Megaspilidae	Parasitoid
Diapriidae	Parasitoid
Figitidae	Parasitoid

**Table 6:** Phase I and Phase II WSU evolutionary participatory breeding (EPB) populations grown by APROSANAMY in Cañar province, Ecuador.

Phase I		Phase II	
Pedigree		Pedigree	
Female	Male	Female	Male
Colorado 407D	3964	Pison	3964
Titicaca	3964	Japanese Strain	3964
Titicaca	Pasankalla	QQ74	3964
Colorado 407D	Pasankalla	23TES	3964
Titicaca	Cica	Titicaca	MisaMisa
Puno	Pasankalla	QQ74	MisaMisa
		Puno	Pasankalla
		Colorado 407D	Pasankalla
		Kaslaea	INIAP Tunkahuan
		Titicaca	Cica

**Table 7:** Timeline of APROSANAMY and WSU collaboration, including dates, locations, and activities.

Planting	Date	Location	Elevation (m a.s.l.)	Activity
	2015	Cañar Comunidad San Rafael (2°32′55.2″ S 78°57′07.0″ W)	3073	- Initiation of APROSANAMY and WSU collaboration
Phase I	23 May 2016	Molobog Chico (2°36′27.252″ S 78°52′48.432″ W)	3203	- Kañari planting ceremony (Figure 5A) - Field preparation, plowing (Figure 5B) - Planting of barley populations with WSU students
		La Posta (2°36′27.252″ S 78°52′48.432″ W)	2956	- Planting of quinoa and barley populations with WSU students (Figure 5C)
	18 March 2017	La Posta	2956	- Leonardo Hinojosa and Dan Packer (WSU) visited second cycle of planting populations
Phase II	22 May 2018	La Posta	2956	- Nicolas Pichazaca planted 10 F_2_ populations (Table 6)
Phase I, II and Pure Line Selection strategy	2 February 2019	La Posta	2956	- Leonardo Hinojosa and Nicolas Pichazaca increased seed from Puno/Pasankalla (Phase I) - They planted F_3_ population (Titicaca/CICA), (Titicaca/MisaMisa) from Phase II - They planted 60 F_6_ lines of (Cahuil/PI 510534) (Phase III)
	15 October 2019	La Posta	2956	- Kevin Murphy, Nicolas Pichazaca, and Cristina Ocaña Gallegos planted 37 quinoa lines
